# High rate of completion for weekly rifapentine plus isoniazid treatment in Chinese children with latent tuberculosis infection—A single center study

**DOI:** 10.1371/journal.pone.0253159

**Published:** 2021-06-11

**Authors:** Heng Yang, Yang Yang, Zhi-dong Hu, Lu Xia, Xu-hui Liu, Xin Yu, Jia-ye Ma, Tao Li, Shui-hua Lu

**Affiliations:** 1 Shanghai Public Health Clinical Center, Fudan University, Shanghai, China; 2 The Affiliated Infectious Hospital of Soochow University, Soochow University, Suzhou, Jiangsu, China; Chinese Academy of Medical Sciences and Peking Union Medical College, CHINA

## Abstract

Three months of weekly rifapentine plus isoniazid (3HP) is a short course regimen for latent tuberculosis infection treatment with satisfied safety and efficacy. However, research on its use in children is limited. In this study, we evaluated the completion rate and safety of the 3HP regimen among children in China. Participants aged 1–14 years receiving 3HP for TB prevention at Shanghai Public Health Clinical Center were followed from December 2019 to November 2020 to evaluate the safety and completion rate of the treatment. Thirty-one children were eligible for inclusion, but five were excluded from the analysis (three were treated with a lower than recommended dose, and two were lost to follow-up). Of the 26 children included in the analysis, the treatment completion rate was 100%. Adverse drug reactions (ADRs) were reported in 38.5% (10/26) of the patients. The most common ADRs were gastrointestinal symptoms (19.2%,5/26), and all ADRs were rated as Grade 1. The 3HP regimen has a high completion rate, and it seems well tolerated in our study population. However, further randomized controlled clinical trial with larger sample size are warranted.

## Introduction

It is estimated that there are 1.93 billion individuals across the globe with latent tuberculosis infection (LTBI), accounting for about one quarter of the world population [1]. Studies suggest that active tuberculosis (TB) will develop in 5–15% of those with LTBI during their lifetimes [2]. The greatest risk of developing active TB is within 2 years after infection [3]. Therefore, preventive treatment for individuals at high risk of progression to active disease is essential for successful control of TB [4]. It is reported that children may benefit more than adults from treatment of LTBI [5].

A 3-month treatment regimen termed 3HP, comprising a once-weekly high-dose of rifapentine plus isoniazid for a total of 12 doses, has become popular for LTBI treatment due to its shorter course and improved completion rate with similar efficacy compared with other recommended LTBI regimens, including isoniazid treatment for 6–12 months, 1 month regimen of daily rifapentine plus isoniazid,4 months of daily rifampicin alone [6, 7]. The 3HP protocol is included in the World Health Organization (WHO) guidelines for the preventive treatment of LTBI and can be used in children, adults, and pregnant women [7, 8].

Previous research has focused on the safety and protection efficacy of 3HP in adults. The main adverse drug reactions (ADRs) for this regime in adults include systemic drug reactions, hepatotoxicity, and gastrointestinal (GI) symptoms. However, few studies have investigated the safety and completion rate of the 3HP regimen in children [5, 9, 10]. Therefore, we conducted this study to evaluate the safety and completion rate of the 3HP regimen among children in China.

## Methods

### Trial design and outcomes

This is a clinical study initiated by researchers. Participants were treated with the 3HP regimen for prophylaxis to reduce the risk of progression of LTBI to active TB. The primary end points were the occurrence of ADRs and the treatment completion rate. The ADR ratings were based on the Common Terminology Criteria for Adverse Events (CTCAE 5.0) [11]. The levels of ADRs ranged from Grade 1 to Grade 5: Grades 1–2 required close attention but not necessarily clinical intervention, and Grades 3–5 required clinical intervention [11]. The correlation between an ADR and treatment was evaluated according to the Naranjo score [12]. This study was approved by the ethics committee of Shanghai Public Health Clinical Center (2019S02903). All of the participants’ guardians provided written informed consent.

### Study participants and intervention

The participants were treated in the Tuberculosis Clinic of the Shanghai Public Health Clinical Center from December 2019 to November 2020. The inclusion criteria were: 1) 1–14 years of age; 2) the child’s guardian provided voluntary written informed consent; 3) positive tuberculin skin test (TST) or interferon-gamma release assay (IGRA); and 4) no evidence of active TB. The exclusion criteria were: 1) indicated resistance to isoniazid or rifapentine; 2) potential interactions between the child’s routine medication and isoniazid or rifapentine; 3) allergy to isoniazid or rifapentine; 4) underlying liver disease; 5) human immunodeficiency virus infection; 6) any other conditions that the researchers deemed to make the child unsuitable for the study.

T-Spot. TB ELISpot (T-Spot; Oxford Immunotec; Abingdon, UK) was used as IGRA. TB-PPD (50 IU/mL) (Beijing Xiangrui Biologicals Co.Ltd) was used as TST. During the 48-to-72-hour period after the TST, an induration diameter of >5 mm, local blister, necrosis or lymphangitis was considered as a positive result.

Extrapulmonary TB was excluded for all the participants according to their symptoms and physical examination by the chief physician. When participants had any special symptoms or signs, radiological examination or other examinations would be performed to determine whether it is extrapulmonary TB.

WHO recommends Children aged < 5 years who are household contacts of people with bacteriologically confirmed pulmonary TB and who are found not to have active TB on an appropriate clinical evaluation or according to the national guidelines should be given TB preventive treatment even if LTBI testing is unavailable in 2020. Accordingly, those who less than 5 years old without IGRA positive results are met the inclusion criteria [7].

From December 2019 to March 2020, the 3HP regimen was provided to participants according to the 2018 WHO guidelines [13]: weekly doses of rifapentine (in children aged 2–14 years, 900 mg for those weighing > 50.0 kg, 750 mg for those weighing 32.1–50.0 kg, 600 mg for those weighing 25.1–32.0 kg, and 450 mg for those weighing 14.1–25.0 kg) and isoniazid (15 mg/kg for those aged ≥ 12 years, 25 mg/kg for those aged 2–11 years) for a total of 12 weeks. From April 2020 to November 2020, 3HP regimen was provided to children aged 2–14 years according to the updated 2020 WHO guidelines [7]: weekly doses of rifapentine (300 mg for children weighing 10–15 kg, 450 mg for those weighing 16–23 kg, 600 mg for those weighing 24–30 kg, and 750 mg for those weighing ≥31 kg) and isoniazid (300 mg for those weighing 10–15 kg, 500 mg for those weighing 16–23 kg, 600 mg for those weighing 24–30 kg, and 700 mg for those weighing ≥31 kg) for a total of 12 weeks.

### Examinations

Baseline data were collected on each participant, including their clinical and demographic data, complete blood counts (CBC), and blood tests of liver and kidney function before starting treatment. The above blood tests were taken in the first, fourth, eighth, and twelfth week to check for ADRs. According to the CTCAE 5.0, treatment would be stopped if a participant experienced Grade 3–5 ADRs. Participants with Grade 1/2 ADRs would be closely observed without discontinuing the treatment. The Naranjo score was calculated to determine whether an ADR was caused by the treatment.

### Statistical analysis

Continuous variables were reported as medians and interquartile ranges (IQR). Categorical variables were reported as counts and percentages. The performance of the 2018 and 2020 regimen was compared using the Wilcoxon signed-rank test. The proportion comparison of 1-sample,1-sided was used to assess the power of completion rate and ADRs incidence with other reported study. P values ≤0.05 were considered statistically significant. Data analyses were performed with statistical software (Microsoft Excel for Windows 10 (Microsoft, Redmond, WA, USA), GraphPad Prism version 8 (GraphPad Software, San Diego, CA, USA) and R software version 4.0.3).

## Results

### Study participants and treatment regimens

A total of 31 children with LTBI were enrolled in the trail at the Tuberculosis Clinic of Shanghai Public Health Clinical Center from December 2019 to November 2020. Five children were excluded from the analysis for the following reasons: three were provided with a lower than recommended dosage, and two were lost to follow-up. After these exclusions, we analyzed the data of 26 participants. These 26 children were either TST or IGRA positive and all ruled out active tuberculosis. There are 26 subjects detailed clinical information including tuberculosis infection results, chest radiography result and reason for eligibility (S1 Table) All of the 26 participants took the medications according to the WHO guidelines and attended follow-up visits on a regular basis. The demographic and clinical characteristics of the study participants are shown in Table 1. Of the 26 children, 18 (69.2%) had close contact with individuals with confirmed pulmonary TB; four (15.4%) were to initiate anti-tumor necrosis factor treatment or immunosuppressive drugs; one (3.8%) had been diagnosed with chronic kidney disease and was receiving glucocorticoid therapy; and three (11.5%) had fevers of unknown origin and positive IGRA results.

**Table 1:** Clinical and demographic characteristics of children in the 3 months of weekly rifapentine and isoniazid for tuberculosis preventive treatment (N = 26).

Characteristic	
Age (years), median (IQR)	5 (4–7.75)
Sex, n (%)	
Male	16 (61.5)
Female	10 (38.5)
Age group, n (%)	
Age 1–4 years	10 (38.5)
Age 5–11 years	14 (53.8)
Age 12–14 years	2 (7.7)
Body mass index (kg/m^2^), median (IQR)	17.2(14.1–17.6)
Reaction size of tuberculin skin test, n (%)	
<5mm	3 (11.5)
5-14mm	10 (38.5)
≥15mm	6 (23.1)
Unknown	7 (26.9)
IGRA result, n (%)	
Positive	24 (92.3)
Negative	1(3.8)
Unknown	1 (3.8)
TST(+) and IGRA (+)	14 (53.8)
TST (+) and IGRA (-)	1(3.8)
IGRA (+) and TST (-)	3 (11.5)
Reason for eligibility, n (%)	
TB close contact in household	7 (26.9)
TB close contact at school	11 (42.3)
Initiating anti-TNF treatment or immunosuppressive drug	4 (15.4)
Chronic kidney disease	1 (3.8)
Fever of unkown origin	3 (11.5)
History of Bacillus Calmette-Guérin vaccination	26 (100)
Chest radiography result	
Normal	6 (21.4)
Abnormality not related to tuberculosis	2 (7.7)
Hilar lymphadenopathy	10 (38.5)
Other mild atypical lesions	11 (42.3)

Abbreviations: IGRA, interferon-gamma release assay; IQR, interquartile range; TB, tuberculosis; TNF, tumor necrosis factor.

**Fig 1:**
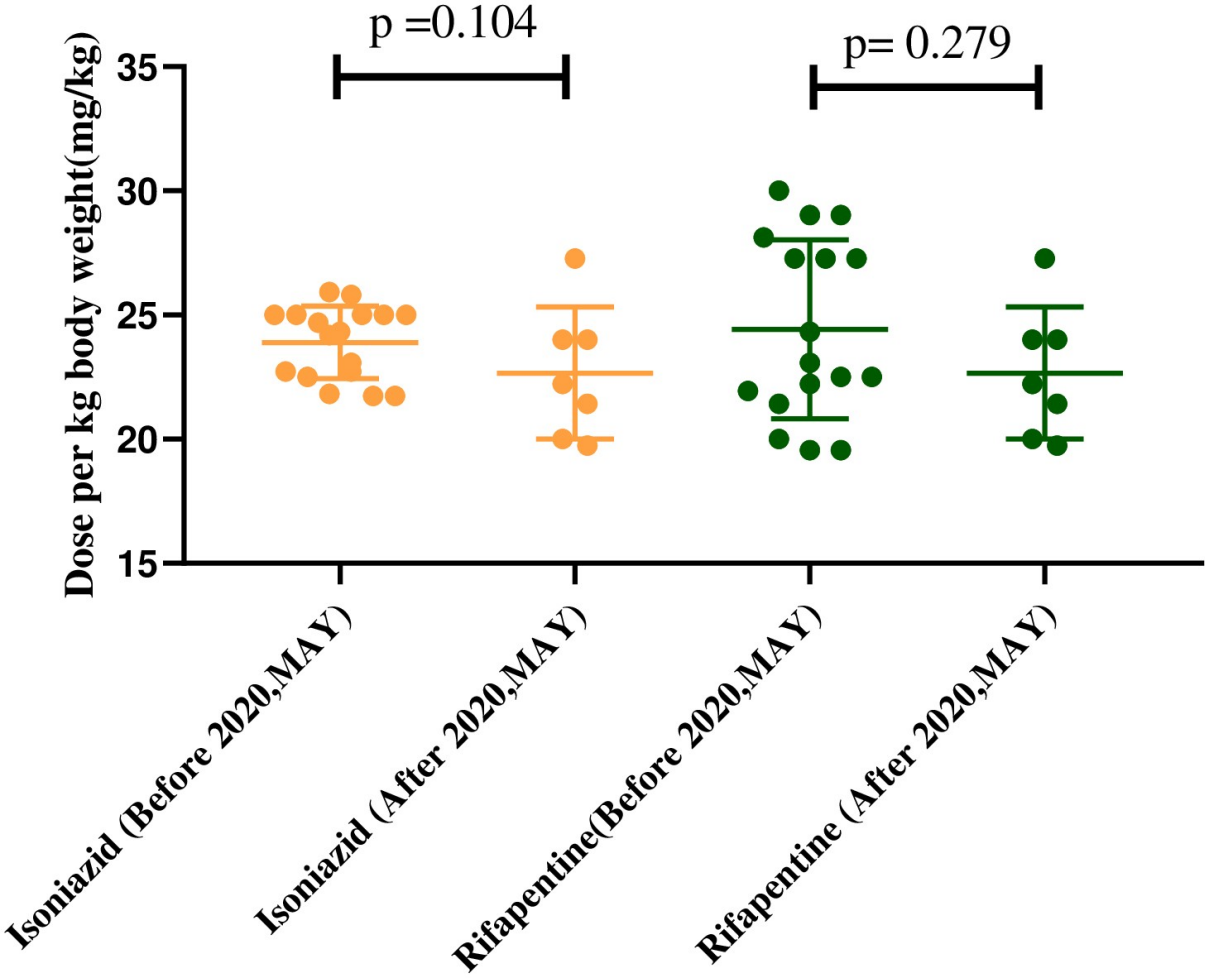
Dose of anti-tuberculosis per kilogram of body weight (24 children).

As described above, from December 2019 to March 2020, the drug dosages were based on the 2018 WHO guidelines, and from April 2020 to November 2020, they were based on the updated 2020 WHO guidelines. Fig 1 shows the drug dose per kilogram of body weight for children 1–12 years of age. The median (interquartile range) isoniazid doses of per kg body weight in group receiving the 2018 regimen and the 2020 regimen were 24.32 mg (22.61–25.00mg) and 22.22mg (20.00–24.00mg) respectively (p = 0.104). The rifapentine doses of per kg body weight in these two groups were 23.08mg (21.69–27.70mg) and 22.22mg (20.00–24.00mg) respectively (p = 0.279).

### Treatment completion and adverse drug reactions

All of the study participants (26/26) completed the treatment. Among them, 38.5% (95% confidence interval, 18.4% to 58.5%) experienced at least once ADRs. All ADRs were classified as Grade 1, no serious adverse events occurred during treatment. GI symptoms were the most frequently reported ADRs (19.2%,5/26), followed by flu-like symptoms and cutaneous reactions (11.5%,3/26). 3.8% (1/26) participant had both flu-like symptoms and a cutaneous reaction. All the symptoms resolved during or after treatment. No participants experienced signs of hepatotoxicity.

The main manifestations of gastrointestinal dysfunction were abdominal pain and vomiting after taking the trial medication, and they were generally transient and improved within a day of onset. There were large differences between participants in the frequency of gastrointestinal discomfort. 11.5% (3/26) had occasional gastrointestinal discomfort after taking the trial medication, and 3.8% (1/26) had nausea almost always after taking rifapentine, abdominal pain, and vomiting.

11.5% (3/26) experienced flu-like symptoms including dizziness, sneezing, and/or fever that lasted several days but resolved before the next dose of study medication. 3.8% (1/26) developed a local rash, and 7.7% (2/26) experienced skin pigmentation. All symptoms disappeared after treatment completion.

3.8% (1/26) had a Naranjo score of 9, indicating that the ADRs (GI symptoms) was definitely drug related; in two participants (7.7%,2/26), the ADR was probably drug related; in six participants, the ADRs was possibly drug related; and in one participant (3.8%,1/26), it is unlikely that the ADR was caused by the treatment (Table 2).

**Table 2:** Treatment outcomes and adverse drug reactions during 3 months of weekly rifapentine and isoniazid for tuberculosis preventive treatment (N = 26).

	(N = 26)
Treatment completion, n (%)	
Completed	26 (100)
Not completed	0 (0)
Adverse drug reactions (ADRs), n (%)	
AnyADR	10 (38.5)
Serious ADR	0 (0)
Flu-like symptoms^a^	3 (11.5)
skin rash (local)	1 (3.8)
skin pigmentation	2 (7.7)
Gastrointestinal symptoms	5 (19.2)
Leukopenia	1 (3.8)
Attributable to the medication ^b^(n,%)	
Definite	1 (3.8)
Probable	2 (7.7)
Possible	6 (23.1)
Unlikely	1 (3.8)
Severity of the ADR, n (%)	
Grade 1	10 (38.5)

^a^ Presence of fever, dizziness, sneezing or other flu-like symptoms.

^b^ We determined whether the ADRs were attributed to the medication using the Nanranjo algorithm. Total score ≥9 = definite; 5–8 = probable; 1–4 = possible; ≤0 = unlikely [12].

Abbreviation: ADR, adverse drug reaction.

## Discussion

This is the first study evaluating the completion rate and tolerability of the 3HP regimen among children in China. The results have illustrated three major implications. First, the participants in the study were well-tolerated to the 3HP regimen. We reported 38.5% (10/26) of participants having ADRs, with all ranking as Grade 1. GI symptoms were the most frequently reported ADRs (5 /26). Except for the ADRs, there was no other peculiar clinical manifestations during and after 3HP treatment. We compared this study with the one also evaluating 3HP regimen in children (ADRs incidence, 8%,43/539) which has the largest sample size [5], and the proportion comparison of 1-sample,1-sided was used. It turned out that the power of ADRs incidence was 99% in our research, which reveals ADRs incidence was higher. Meanwhile, the incidence of ADRs in our study was lower and the ADRs were milder than those reported in other studies in China. One study of individuals with silicosis receiving the 3HP regimen found that 70.4% (169/240) of participants experienced adverse events and 7.9% (19/240) experienced grade 3 or 4 adverse events [14]. Another study of 3HP treatment among older adults was terminated early due to an unexpectedly high incidence of adverse events [15]. This may have been related to their older age and comorbidities. Huang et al. [16] assessed the impact of age on the outcome of the 3HP regimen. They found that older adults (aged ≥65 years) had a higher risk of grade ≥3 ADRs and a higher frequency of ADRs than middle-aged (35–65 years) and younger (18–35 years) participants. Furthermore, it has been reported that 2–10% of individuals experienced systemic drug reactions [17], defined as either of the following: (1) hypotension, urticaria, angioedema, acute bronchospasm, or conjunctivitis; or (2) >4 flu-like symptoms, with at least one rated grade 2 or higher^17^. None of the participant in our study met these criteria.

The participants showed high completion rate to the 3HP regimen. All pariticpants completed the 12 doses of drugs in accordance with the study protocol. The completion rate in our study was 100% (26/26). As compared to the published studies reported the completion rate of 3HP in children (62%-88%), the power of our study to identify such completion rates was as high as 100%. The low incidence and low grade of the ADRs observed in our study might be a major reason for the high completion rate. Further, the high adherence ascribed to the motivated parents also should be counted. As most children belonged to the high-risk population defined by the WHO guidelines [13], their parents were concerned about their risk of progressing active tuberculosis and thus strictly adhered to doctors’ instructions.

Further studies are warranted to investigate fixed-dose combinations and child-friendly dosage forms. Three children were excluded due to receiving insufficient doses of the drug. Such dosing errors are not rare in the treatment of LTBI, as demonstrated in a previous study that reported dosing errors in 4.5% (68/1520) of participants [17]. The 3HP regimen in children requires different dosing based on age and weight. It is inconvenient for physicians to calculate the correct dosage for each child. In our study, there was no statistically significant difference in the recommended dosage of 3HP per kilogram of body weight between children treated according to the 2020 WHO guidlines and those treated according to the 2018 WHO guidelines. In general, the 2020 guidlines are more convenient in clinical practice than the 2018 guidlines. Moreover, it is difficult for children to take 5–12 tablets of anti-TB drugs per each dose; further, children under 5 years have difficulty swallowing capsules. The development of soluable fixed dose combinations would reduce pill burden, improve prescription accuracy, maintain good compliance, and improve the patients’ treatment experience.

There are several limitations in our study. First, the sample size was too small to provide strong evidence for estimating the occurrence of rare ADRs. Therefore, our results on safety should be interpareted with caution. Second, this was single-arm clinical study and the performance of the 3HP regimen was not compared with the other regimes. Although, according to the current available evidence [5, 18], it acts as effective as regimens for LTBI treatment in children.

In conclusion, as we know, this is the first study evaluated the completion rate of 3HP regimen among children with LTBI in China. The findings indicate a good adherence and tolerence in our study population. Further randomized controlled clinical trials with larger sample sizes are needed to verify our results. In order to reduce pill burden and improve perscription accuracy, fixed dose combinations and child-friendly dosage forms should be explored.

## Supporting information

S1 Table Tuberculosis infection results, chest radiography result and reason for eligibility of 26 subjects. (DOCX)
