# Supplementary material for: High rate of completion for weekly rifapentine plus isoniazid treatment in Chinese children with latent tuberculosis infection—A single center study
Source: PLoS One. 2021 Jun 11;16(6):e0253159. doi: 10.1371/journal.pone.0253159 (PMC8195436; doi:10.1371/journal.pone.0253159)
Supplement: S1 Table — (DOCX) [file pone.0253159.s001.docx]

**S1 Table: Tuberculosis infection results, chest radiography result and reason for eligibility of 26 subjects.**

|  | TST | IGRA | Reason for eligibility | Chest radiography result |
| --- | --- | --- | --- | --- |
| Case 1 | 5-14mm | Negative | 1TB close contact in household | Normal |
| Case 2 | Unknown | Positive | TB close contact at school | Hilar lymph node |
| Case 3 | <5mm | Positive | 4Initiating anti-TNF treatment | Other possible tuberculosis-related abnormality |
| Case 4 | Unknown | Positive | 2TB close contact in household | Hilar lymph node |
| Case 5 | ≥15mm | Positive | 3TB close contact in household | Other possible tuberculosis-related abnormality |
| Case 6 | Unknown | Positive | 3Initiating immunosuppressive drug | Normal |
| Case 7 | 5-14mm | Positive | 4TB close contact in household | Hilar lymph node |
| Case 8 | ≥15mm | Positive | TB close contact at school | Other possible tuberculosis-related abnormality |
| Case 9 | Unknown | Positive | TB close contact at school | Other possible tuberculosis-related abnormality |
| Case 10 | ≥15mm | Positive | 5TB close contact in household | Other possible tuberculosis-related abnormality |
| Case 11 | Unknown | Positive | 6TB close contact in household | Hilar lymph node; Other possible tuberculosis-related abnormality |
| Case 12 | 5-14mm | Positive | TB close contact at school | Normal |
| Case 13 | 5-14mm | Positive | TB close contact at school | Hilar lymph node |
| Case 14 | ≥15mm | Positive | TB close contact at school | Other possible tuberculosis-related abnormality |
| Case 15 | 5-14mm | Positive | TB close contact at school | Other possible tuberculosis-related abnormality; Hilar lymph node |
| Case 16 | 5-14mm | Positive | 2Initiating immunosuppressive drug | Other possible tuberculosis-related abnormality |
| Case 17 | ≥15mm | Unknown | TB close contact at school | Hilar lymph node |
| Case 18 | Unknown | Positive | TB close contact at school | Hilar lymph node |
| Case 19 | Unknown | Positive | TB close contact at school | Abnormality not related to tuberculosis |
| Case 20 | 5-14mm | Positive | Fever of unkown origin | Other possible tuberculosis-related abnormality |
| Case 21 | 5-14mm | Positive | Fever of unkown origin | Abnormality not related to tuberculosis |
| Case 22 | 5-14mm | Positive | TB close contact at school | Normal |
| Case 23 | 5-14mm | Positive | 7TB close contact in household | Hilar lymph node |
| Case 24 | <5mm | Positive | 1Initiating immunosuppressive drug | Normal |
| Case 25 | <5mm | Positive | 1Chronic kidney disease | Normal |
| Case 26 | ≥15mm | Positive | Fever of unkown origin | Other possible tuberculosis-related abnormality; Hilar lymph node |
